# Atopic dermatitis and psychosocial comorbidities – What’s new? 

**DOI:** 10.5414/ALX02174E

**Published:** 2020-11-06

**Authors:** Paula Kage, Julia Zarnowski, Jan-Christoph Simon, Regina Treudler

**Affiliations:** Department of Dermatology, Venereology and Allergology, Leipzig Interdisciplinary Center for Allergology – LICA-CAC, University of Leipzig, Germany

**Keywords:** atopic dermatitis, atopic eczema, depression, anxiety, attention deficit hyperactivity disorder, schizophrenia, anorexia, obsessive compulsive disorder

## Abstract

Atopic dermatitis (AD) is a chronic inflammatory disease. During the last years, researchers have focused on a variety of associated comorbidities, especially psychosocial disease. This article aims at giving an overview over recent data. A systematic literature research was performed in PubMed including data from the time period January 1, 2018 until March 1, 2020. Patients with AD frequently suffer from cocomitant depression, anxiety, and attention deficit hyperactivity disorder. There is less evidence about the relation between AD and schizophrenia, eating disorder, and obsessive compulsive disorder. There is still great need for research in the connection between AD and psychosocial disease, particularly about the pathogenesis and the influence of new therapies.

[Table Abbreviation]

## Introduction 

Well-known comorbidities of atopic dermatitis (AD) are allergic bronchial asthma, allergic rhinoconjunctivitis, and food allergy [25]. This work aims to provide an overview of the current data on psychosocial comorbidities in AD. 

## Materials and methods 

**Figure 1.**
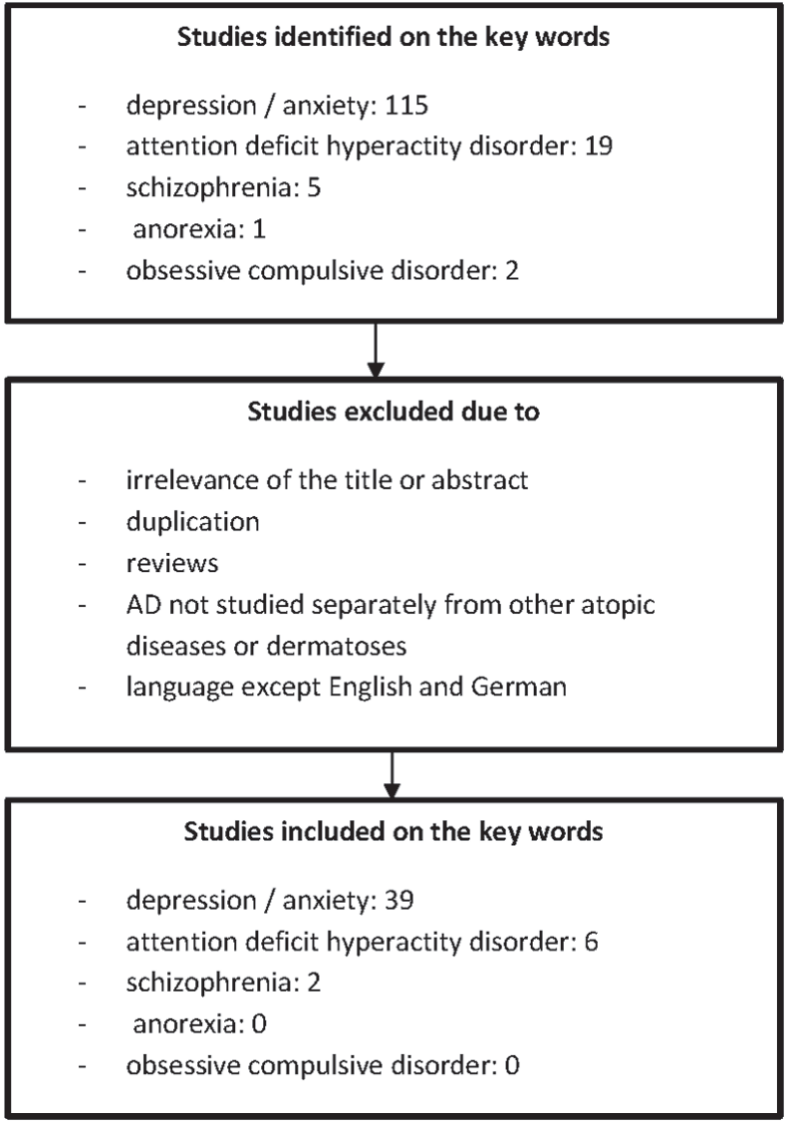
Flowchart of the selection process of included studies.

PubMed was searched for the terms atopic dermatitis/eczema, depression, anxiety, attention deﬁcit hyperactivity disorder, anorexia, obsessive compulsive disorder, schizophrenia. Data from the period January 1, 2018 to March 1, 2020 were included. After a systematic literature search, 47 studies were identified, including cross sectional studies (CS), case control studies (CC), and randomized, placebo-controlled studies. The number of patients examined ranged from 9 to 2.2 million patients (Figure 1). 

## Depression and anxiety (Table 1) 

In worldwide studies, patients with AD had a significantly higher prevalence of depression (9.3 – 44.3%) compared to those who were not affected [2, 11, 12, 17, 18, 19, 22, 28, 30, 46, 47, 48]. The risk was 1.14- to 2.86-fold increased [11, 12, 13, 29, 33]. 

Patients with AD were also significantly more likely to suffer from anxiety [18] (prevalence 3.31 – 26.2% [2, 12, 28], risk increase of 1.74- to 3-fold [12, 29, 45]). 

When depression and anxiety were recorded together, the prevalence rates were 40 – 50.2% [37, 39, 40, 41, 42] with a 1.11- to 2.34-fold higher risk of occurrence [5, 37, 40]. 

Many studies have shown a positive correlation between the severity of AD and the severity of mental illness and the use of antidepressants and anxiolytics [1, 6, 12, 14, 18, 19, 30, 31, 33, 36, 37, 38, 39, 41, 44, 45]. Only four studies could not show any differences in depressive symptom scores between patients with AD and controls [1, 16, 34, 47]. 

In Leipzig, it was shown that the risk of depression in AD patients is comparable to the risk of depression in cancer patients. In tumor therapy, due to the high incidence of psychosocial diseases, additional support from psycho-oncologists has long been established. The risk of anxiety was lower in AD patients than in cancer patients, but higher than in patients with diabetes mellitus or stroke [46]. 

AD patients with employments had significantly higher levels of depression, stress, and anxiety than those who were not working. The stress levels were increased in AD patients who smoke as opposed to non-smoking ones [16]. 

In children with AD, the risk of depression and anxiety disorder was shown to be increased if they had a step-parent [29]. Mothers of children with AD had higher anxiety scores [10]. 

In three studies it could be shown that an adequate therapy of the skin symptoms leads to a reduction of the symptoms of anxiety and depression [7, 15, 48]. An exposure-based cognitive behavioral treatment showed a significant reduction in anxiety but no significant differences in depressive symptoms. Training the mothers of children with AD significantly reduced the anxiety levels of the mothers [50]. 

Little is known about the direct causal relationship between depression/anxiety and AD. In the period investigated, there were only two studies that dealt with the physical pathophysiology of the relationship. A significantly reduced serotonin level has been shown in patients with severe AD [24]. In men with extrinsic AD, blood cortisol levels were inversely correlated with anxiety scores [48]. 

In the study by Singh et al. [43] in medical consultations with both dermatologists and other specialists, only 1.2% of patients with AD were screened for depression. In men this was carried out even less than in women (0.8 vs. 2.4%). There was no significant difference in the frequencies of screening tests for depression in AD patients with regard to therapy intensity. 

In summary, the connection between anxiety, depression, and AD has been strengthened over the past 2 years. Nevertheless, many questions regarding the influencing factors and the causes remain unanswered so that further research is necessary. 

## Attention deficit hyperactivity disorder (Table 2) 

For patients with AD, especially children, an increased risk of attention deficit hyperactivity disorder (ADHD) could be shown. Only for children with AD, also an increased risk of hyperactivity, attention deficit, and impulsivity was seen [1, 3, 8, 26, 32, 49]. 

It has been suggested that sleep deprivation due to neurocognitive disorders could be a trigger for attention deficit [3]. The use of antihistamines in children with AD, as well as the current severity of AD symptoms, showed a significant association with increased occurrence of ADHD symptoms [32]. Little is known about the direct causal relationship between ADHD and AD. Children with ADHD and children with AD and ADHD showed a reduced cortisol response to acute stress, but children with AD only did not [8]. Siblings of patients with ADHD were at increased risk of developing AD, asthma, allergic rhinoconjunctivitis, and other atopic diseases. It is believed that the same risk factors for developing atopic diseases exist as for ADHD [9]. 

## Schizophrenia (Table 3) 

Patients with AD had an increased risk of psychotic events (OR 1.20; p = 0.009) and hallucinations (OR 1.24; p = 0.002) compared to the general population [4]. Adult patients with AD had an increased risk of being hospitalized for mental illness (OR 1.78), but children did not (OR 0.68). Adults and children with AD showed an association with anxiety disorders and developmental disabilities. An association between AD and mood disorders, schizophrenia, addictions, personality disorders, adjustment disorders, ADHD, or behavioral disorders was only found in adults but not in children. Patients with AD who were hospitalized for mental illness were more likely to be younger, of Asian or African American descent, higher income, and multiple chronic illnesses [23]. 

## Anorexia and obsessive-compulsive disorder 

No data on anorexia and obsessive-compulsive disorder in AD could be identified for the period investigated. 

## Conclusion 

The research carried out in recent years more and more supports the connection between AD and psychosocial comorbidities. Due to the frequent presence of these disorders, screening for depression, anxiety, and other mental illnesses should be rigorous. This is necessary so that psychological support can be involved at an early stage if the illness occurs. The consequences of the presence and severity of AD on mental health should be taken into account when making therapy decisions, as adequate therapy can lead to the prevention and alleviation of mental symptoms. Despite the increasingly clear connection between AD and psychosocial comorbidities, many questions about the causal pathophysiology remain unanswered. Therefore, further research in this area is urgently needed. 

## Conflict of interest 

The authors declare that there is no conflict of interest. 

## Funding 

Paula Kage is grateful for funding of the Hautnetz Leipzig/Westsachsen e.V. for research on atopic dermatitis and comorbidities. 

**List of abbreviations Abbreviation:** List of abbreviations.

WHO-5	5-item World Health Organization Well-Being Index
AD	Atopic dermatitis
AESEC	Atopic eczema score of emotional consequences
BASC-2	Behavior assessment system for children 2^nd^ edition
BAI	Beck anxiety inventory
BDI	Beck Depression Inventory-short form
CES-D	Centre of epidemiologic studies-depression scale
CPRS-R	Conners’ parent rating scale
DASS-42	Depression, stress and anxiety scale
DMS-IV	Diagnostic and statistical manual of mental disorders-IV
EASI	Eczema area and severity index
EQ5D-5L	Five-dimension five-level version of the EQ-5D
FPI-R	Freiburg personality inventory
FBB-ADHS	External assessment sheet attention deficit/hyperactivity disorder (Fremdbeurteilungsbogen Aufmerksamkeitsdefizit-/Hyperaktivitätsstörungen)
GAD-7	Generalized anxiety disorder
GT	Gießen Test
HAM-A	Hamilton anxiety rating scale
HAMD	Hamilton depression rating scale
ICD	International statistical classification of diseases and related health problems
ISI	Insomnia severity index
K-6	Kessler-6 index
LSNS	Lubben social network scale
MADRS	Montgomery Åsberg depression rating scale
MPT	Munich personality test
NEO-FFI	Neo-five factor inventory
PHQ-2	Patient health questionnaire-2
POEM	Patient oriented eczema measure
QoLPAD	Quality of life in parents of children with atopic dermatitis
QOLI	Quality of life inventory
QPE	Questionnaire for psychotic experiences
SCORAD	SCORing atopic dermatitis
SCARED	Screen for child anxiety related emotional disorders
SCS	Self-consciousness-scale
SF-8	Short form health survey 8
SF-12	Short form health survey 12
SMFQ	Shortened mood and feelings
STAI	State-trait anxiety inventory
SDQ	Strengths and difficulties questionnaire
TEG-II	Tokyo University Egogram II
TSST-C	Trier social stress test for children
VADRS	Vanderbilt ADHD diagnostic rating scale

**Table 1.** Studies on depression and anxiety.

Author	Study design	Parameter	Number of patients, age in years (y)	Country	Conclusion
[19]	CS	Natural language processing, IGA	1,231 with AD ≥ 18 y	USA, K, F, D, I, E, UK	Depressive feelings, anxiety and hopelessness are more common in moderate/severe AD than in mild AD.
[24]	Pro, CC	Serum serotonin levels, Hanifin & Rajka, SCORAD, MADRS	31 with AD 14 controls Mean: 41 y	Po	All pat. with SCORAD > 50 had depression. Serotonin levels significantly lower in pat. with severe AD. Inverse correlation between serotonin level and depression score.
[48]	Pro, CC	Total IgE, cortisol and testosterone levels, HAMD, SCORAD	56 with AD 49 controls	Israel	All pat. with AD had an elevated score for depression. After adequate treatment of the skin, the depression values improved in all pat. with intrinsic AD and in men with extrinsic AD. In men with extrinsic AD, blood cortisol levels correlated inversely with the SCORAD and HAMD scores.
[16]	Pro, CC	Medical history, questionnaires, depression, DASS-42	75 with AD 75 controls 18 – 41 y	Turkey	No difference between the scores for depression and anxiety in pat. with AD and controls (p > 0.05). Working AD pat. showed significantly higher levels of depression and anxiety than non-employed pat. (p < 0.05). The stress values were elevated in smoking as opposed to non-smoking AD pat.
[46]	CS	CES-D, GAD-7, LSNS, SF-8	9,481; thereof 372 with AD ≥ 18 y	D	Pat. with AD showed higher scores for depressive symptoms (9.3 vs. 6.3%; p < 0.001) and anxiety (8.4 vs. 5.6%, p < 0.001). The risk of depression in pat. with AD (OR 1.5; p = 0.031) was comparable to the risk in cancer pat. (OR 1.6; p = 0.001). The risk of anxiety was higher in AD (OR 1.5; p < 0.049) than in diabetes mellitus (OR 1.2) and stroke (OR 1.4), but lower than in cancer pat. (OR 1.9).
[35]	CS	ICD-9, ICD-10	2.2 million thereof 62.849 with AD > 6 y	E	The frequency of diagnosis of anxiety and agitation was higher in pat. with severe AD than in the normal population In severe AD, the prevalence of anxiety was 79.7% (6 – 12 y), 65.8% (13 – 18 y) and 67.3% (> 18 y).
[22]	CS	Medical history, EASI, SCORAD, IGA, DLQI, PGA, NRS	612 with AD 42.6 ± 14.2 y	D	Pat. with AD described depression more often than the normal population (10 vs. 7.7%).
[43]	CS	ICD-9, medical history, frequency of depression screenings	9,345 with AD	USA	During medical consultations (with dermatologists and other specialists), 1.2% of AD pat. were subjected to a screening test for depression. This was done less often in men than in women (0.8 vs. 2.4%). No significant difference on the frequency of the screening test in AD pat. in terms of therapy intensity.
[6]	CS	IGA, EASI, SCORAD, POEM, HADS, DLQI, medication	18 – 65 y	K, F, D, I, E, UK	Pat. with severe AD had an increased prevalence of psychological comorbidities compared to those with mild to moderate AD (p < 0.001).
[33]	CS	ICD-10	526,808 with AD 2,569,030 controls ≥ 18 y	UK	AD was associated with an increased incidence of newly onset depression (HR, 1.14) and anxiety (HR 1.17). A strong effect of AD on depression with increasing severity of AD was observed: HR compared to healthy subjects: mild, 1.10; moderate, 1.19; and heavy, 1.26. This dependence on severity was weaker in relation to anxiety disorders: HR compared to healthy subjects: mild, 1.14; moderate, 1.21; and severe, 1.15.
[17]	CS	ICD-10	656 with AD 52.1 ± 17.9 y	UK	The prevalence of depression was 10.8%.
[44]	CS	Questionnaires, 5-item World Health Organization Well-Being Index (WHO-5)	34,313 thereof 4,175 with AD	S	Adults with mild AD had an increased risk of major depression (RR 1.78) and anxiety (RR 1.97). The risk in severe AD was even higher for depression (RR 6.22) and anxiety disorders (RR 5.62).
[14]	Pro, double blind, cohort	AD diagnosed by physician, prescriptions	844 with AD 25 ± 16.5 y	UK	The prevalence of having repeatedly prescribed antidepressants was twice as high in AD pat. with high need for topical steroids compared with AD pat. with low need (12 vs. 6%).
[1]	CS	ICD-10	42,641 with AD 139,486 with other dermatosis 0 – 65 y	Ko	The incidence of depression did not differ between pat. with/without AD. In severe AD higher risk of depression (OR = 3.15, p < 0.0001) than in moderate (OR = 1.75). In severe AD significantly higher incidence of ADHD (OR = 1.48), autism spectrum disorders (OR = 1.54), and behavior disorders (OR = 2.88).
[11]	CS	ICD-9, HQ-2, K-6	19,840 ≥ 18 y	USA	Pat. with AD were screened positive for depression more often than controls (44.3 vs. 21.9%) and had a higher risk for it (OR 2.86, p = 0.02); reported more often about depressed mood (OR 2.94; p = 0.04) and anhedonia (OR 2.47; p = 0.05); had a higher risk of feeling hopeless (OR 2.51), restless (OR 2.88), apathetic (OR 3.20), and worthless (OR 3.06). In AD pat. an increased K-6 point value was found for low and middle income, but lower for pat. of African American origin and other/multiple descent.
[15]	Randomized, placebo-controlled, phase 3	EASI, HADS	1,379 with AD 25 – 51 y	USA Europa Asia	Dupilumab improved symptoms of depression and anxiety as measured by HADS compared to placebo (p < 0.001).
[30]	CS	POEM, DLQI, HADS, AESEC	1,189 with AD	F, E, I, UK, D, NL, DK, S, Czech Republic	10% of the pat. with AD showed depressive symptoms. 57% of the pat. with AD showed feelings of impairment from their AD. 88% of the pat. with severe AD stated that they felt restricted in coping with their lives due to the AD. The presence of depressive symptoms correlated with POEM score. Depressive tendencies were found in 1% of the pat. who had no/almost no symptoms vs. 21% with severe symptoms.
[39, 41]	CS	POEM, PO-SCORAD, DLQI, HADS, SF-12, SF-6D	2,893 thereof 602 with AD 52.0 ± 16.3 y	USA	PO-SCORAD and POEM showed a moderate to strong correlation with DLQI and HADS. Pat. with AD had significantly higher anxiety and depression scores compared to controls for HADS (p < 0.03). All pat. with severe AD showed increased HADS values. Of the pat. with AD, 40.0% stated that they had suffered from depression or anxiety in the past year compared to 17.5% of the controls.
[18]	CS	SF-36, HADS, DLQI	1,860 with AD 1,860 controls	F, D, I, E, UK	Depression had a higher prevalence in pats. with AD compared to controls (25.8% in pat. with controlled AD and even higher with 36.2% in pat. with uncontrolled AD compared to 12.9%). Anxiety also had a higher prevalence in pat. with AD compared to controls (31.6% in pat. with controlled vs. 51.7% in pat. with uncontrolled AD compared to 14.4%).
[13]	CS	Questionnaire	917,948 thereof 21,111 with AD	Ko	Pat. with AD had a 2.31-fold higher risk of being diagnosed with depression compared to the normal population.
[38]	CS	PO-SCORAD, POEM, DLQI, HADS, SF-12	3,495 thereof 602 with AD ≥18 y	USA	HADS showed a strong correlation with SF-12 and moderate to weak correlation with PO-SCORAD, POEM, and DLQI.
[10]	CS	BDI, HAM-A	24 children with AD and their mothers 24 controls their mothers	Tunisia	The mothers in the AD group showed higher HAM-A scores but no increased depression scores in the BDI compared to the controls.
[12]	CS	POEM, DLQI, HADS	1,185 thereof 93 with AD 51.81 ± 18.17 y	USA	24.73% of the pat. with AD showed the clinical signs of anxiety compared to 9.20% of the controls (p < 0.001). 13.98% of pat. with AD showed clinical signs of depression compared to 9.20% of controls (p < 0.003). In a comparison of moderate and severe AD, the achievement of an increased depression value was found to be the same (19.54 vs. 19.70%). This was higher than for mild AD (8.84%). Pat. with AD had a 3-fold increased risk of anxiety and a 2.5-fold increased risk of depression.
[20]	CC	ICD-8 to 10	8,602 children with AD 86,602 controls	DK	No significant association between mental illnesses (depression, alcohol addiction, and drug addiction) in the parents and the development of AD in their children.
[28]	CS	POEM, SA-EASI, medical history	287 with AD 18 – 69 y	USA	26.2% of pat. with AD had anxiety in the past, 34.0% had depression.
[21]	Open pilot study cognitive behavioral treatment	SCORAD, BAI, MADRS	9 with AD 42.8 ± 14.2 y	S	Significant reduction in anxiety measured with BAI but no significant difference in depressive symptoms measured with MADRS.
[37, 40]	CS	Medical history, PO-SCORAD, POEM, HADS	8,217; thereof 602 with AD	USA	In AD, increased risk of anxiety and depression compared to controls (OR 2.34, 95% CI 1.91 – 2.87). 40% of the pat. with AD reported suffering from anxiety or depression.
[42]	CS	PO-SCORAD, HADS, DLQI	1,519 with AD 45.7 ± 17.4 y	USA	In moderate/severe AD, significantly higher prevalence of anxiety and depression vs. mild AD (50.2 vs. 27.3%, p < 0.001). Anxiety and depression symptoms were not significantly higher in pat. with uncontrolled vs. controlled AD.
[29]	CS	Medical history, questionnaires	13,275 children; thereof 12.29% with AD ≤17 y	USA	In pat. with AD, higher risk for depression (OR 2.287), anxiety (OR 2.001), and stress (OR 2.013) compared to controls. With a step-mother, children are at a higher risk of depression (OR 3.073), anxiety (OR 3.290), and stress (OR 2.300). With a step-father, children had an increased risk for depression (OR 4.386), anxiety disorder (OR 3.778), and stress (OR 2.542).
[47]	CS	ICD-9	46,647 with atopic diseases 139,941 controls	TW	Pat. with AD alone or AD and allergic rhinitis showed a lower risk of mental illness (OR 0.256; p = 0.031) (OR 0.554; p = 0.018). Pat. with AD and asthma as well as pat. with AD, asthma, and allergic rhinitis showed an increased risk of mental illness (OR 1.723; p < 0.001) (OR 3.702; p = 0.027).
[5]	CS	Medical history, SCORAD, SMFQ	14,197; thereof 3,152 with AD 9 y	S	Children who had ever suffered from AD were shown to have an increased risk of depression or anxiety (OR 1.23; p < 0.01). In children who currently had AD, the risk of depression or anxiety was increased, but not significantly (OR 1.11; p > 0.05).
[2]	CS	Medical history, SF-36	638 with AD 1,268 controls 38.51 ± 12.92 y	Japan	The self-reported prevalence of depression (10.25%) and anxiety (3.31%) was higher in AD pat. compared to controls (p < 0.001). The severity of AD did not have a significant impact on the prevalence.
[50]	Pro, interventional educational program for AD	SCORAD, anxiety measurement by Spielberger	20 mothers of children with AD ≥18 y	South Korea	The scores for anxiety decreased significantly after the training. The mother’s score for anxiety was independent of mother or child age, gender, family history of AD, and onset of symptoms.
[7]	Randomized, double-blind, placebo-controlled, phase 3	IGA, SCORAD, EASI, DLQI, POEM, HADS	325 with AD ≥18 y	Europe	Dupilumab in combination with topical steroids significantly improved AD, quality of life, and symptoms of anxiety and depression compared to topical steroids alone (p < 0.001).
[36]	CS	ICD-9	6,186 with AD	E	26.1% of the pat. took anxiolytics, 22.7% antidepressants. The severe form of AD was associated with depression.
[27]	CS	Questionnaire, EQ-5D	37,578; thereof 677 with AD ≥19 y	Ko	Pat. with AD demonstrated severe psychological stress (p < 0.001), a higher prevalence of depressive mood (p = 0.001), use of psychological counseling centers (p = 0.001), depression (p = 0.002), and suicidal ideation (p < 0.001) significantly more often.
[34]	Blinded, interventional Itch induction	SCORAD, HADS, SCS	23 with AD 23 controls ≥18 y	D	No difference between pat. with AD and the controls with regard to depression and self-confidence in public. No relationship between depression and itch induction.
[45]	CS	Medical history, DSM-5, hospitalization rate, suicide rate, prescriptions	9,656 thereof 1,044 with AD ≥18 y	DK	Significant association between AD and depression (OR 1.92), and diagnosed anxiety (OR 1.74). Significantly more frequent depressive symptoms in AD (OR 2.15). Hospitalization rate not increased compared to the normal population. In the presence of moderate to severe AD, significantly more intake of anxiolytics (OR 1.66) and antidepressants (OR 1.24). In mild AD, only a slight increase in the use of anxiolytics (OR 1.08).
[31]	CS	ICD-8, prescriptions	201,090 men thereof 691 with AD 17 – 20 y	S	Increased risk of antidepressant prescription in AD (OR 1.43; p < 0.001).

CS = cross sectional study; CC = case control study; pro = prospective; pat. = patient; y = years; D = Germany; DK = Denmark; S = Sweden; F = France; I = Italy; K = Canada; Ko = Korea; Po = Poland; E = Spain; TW = Taiwan; UK = United Kingdom; OR = odds ratio; RR = relative risk; HR = hazard ratio

**Table 2.** Studies on ADHD.

Author	Study design	Parameter	Number of patients, age in years (y)	Country	Conclusion
[3]	CS	Conner rating Scale for ADHS	95 with AD 4 – 18 y	Iran	Prevalence of hyperactivity 20%; attention deficit 29.47%. Attention deficit was associated with AD in the cheek area (p = 0.01) and sleep problems (p = 0.01).
[9]	Cohort	ICD-9	20,170 siblings of pat. with ADHS 80,680 controls 15.7 ± 5.3 y	TW	Siblings were at an increased risk of developing AD (RR 1.10), asthma, allergic rhinoconjunctivitis, and other atopic diseases.
[8]	Pro, CC	Cortisol levels in saliva, SCORAD, POEM, FBB-ADHS, TSST-C, ICD-10	42 with AD 34 with ADHS 31 with AD and ADHS 47 controls 6 – 12 y	D	Children with AD showed increased AHDS-like behavior such as inattention, impulsiveness. Children with ADHD as well as children with AD and ADHD showed a reduced cortisol response to acute stress, but children with AD only did not.
[26]	CC	SCORAD, VADRS	17 with AD 18 controls 6 – 17 y	USA	The VADRS screening test was not significantly more positive for ADHD (p = 0.47) or other behavioral disorders (p = 0.23) in children with AD compared to controls. Children with AD showed ADHD-associated behaviors such as inattention.
[32]	Pro, non-interventional	ICD-10, SCORAD, POEM, medical history	154 children 42 with AD 34 with ADHS, 31 with AD and ADHS 47 controls 6 – 12 y	D	Compared to the control group, significantly increased risk for behavioral problems and lower quality of life in patients with AD alone, ADHD alone, or both AD and ADHD. Higher risk for symptoms of ADHD in children with AD compared to the control group. Intake of antihistamines showed significant association with increased incidence of ADHD symptoms (OR1.88). Current severity of AD symptoms with influence on the severity of ADHD symptoms.
[49]	CS	Medical history, DSM-4	2,772 thereof 411 with AD 3 – 6 y	TW	Increased risk for ADHS (OR 4.5) in pats. with AD compared to controls..

CS = cross-sectional study; CC = case control study; pro = prospective; pat. = patient; y = years; D = Germany; TW = Taiwan; OR = odds ratio; RR = relative risk.

**Table 3.** Studies on schizophrenia.

Author	Study design	Parameter	Number of patients Age in years (y)	Country	Conclusion
[4]	CS	Medical history, QPE	6,479 thereof 1,181 with AD ≥ 14 y	Netherlands	Increased risk of psychotic events (OR 1.20; p = 0.009) and hallucinations (OR 1.24; p = 0.002) in AD compared to the general population.
[23]	CS	ICD-9	835 with AD 2,434,703 controls ≥ 5 y	USA	Adults with AD: increased risk of hospitalization due to mental illness (OR 1.78), children not (OR 0.68). These hospitalized pat. were more often younger, of Asian or African-American descent, had a higher income, and several chronic diseases. Adults and children with AD showed an association with anxiety and developmental disorder. An association between AD and mood disorder, schizophrenia, addiction, personality disorder, adaptation disorder, ADHD, or behavioral disorder was only found in adults.

CS = cross sectional; pat. = patient; y = years; OR = odds ratio.
